# The miRNA and mRNA Signatures of Peripheral Blood Cells in Humans Infected with *Trypanosoma brucei gambiense*

**DOI:** 10.1371/journal.pone.0067312

**Published:** 2013-06-27

**Authors:** Smiths Leong, Gustave Simo, Mamadou Camara, Vincent Jamonneau, Jacques Kabore, Hamidou Ilboudo, Bruno Bucheton, Jörg D. Hoheisel, Christine Clayton

**Affiliations:** 1 Division of Functional Genome Analysis, Deutsche Krebsforschungszentrum (DKFZ), Heidelberg, Germany; 2 Department of Biochemistry, University of Dschang, Dschang, West Cameroon; 3 Programme National de Lutte contre la Trypanosomiase Humaine Africaine en Guinée, Conakry, Guinée; 4 Centre international de recherche-développement sur l’elevage en zone subhumide (CIRDES), Bobo-Dioulasso, Burkina Faso; 5 Institut de Recherche pour le Développement, Unité mixte de recherche 177 (UMR-177), Campus International de Baillarguet, Montpellier, France; 6 Zentrum für Molekulare Biologie der Universität Heidelberg, DKFZ-ZMBH Alliance, Heidelberg, Germany; University of California, Riverside, United States of America

## Abstract

Simple, reliable tools for diagnosis of human African Trypanosomiases could ease field surveillance and enhance patient care. In particular, current methods to distinguish patients with (stage II) and without (stage I) brain involvement require samples of cerebrospinal fluid. We describe here an exploratory study to find out whether miRNAs from peripheral blood leukocytes might be useful in diagnosis of human trypanosomiasis, or for determining the stage of the disease. Using microarrays, we measured miRNAs in samples from *Trypanosoma brucei gambiense*-infected patients (9 stage I, 10 stage II), 8 seronegative parasite-negative controls and 12 seropositive, but parasite-negative subjects. 8 miRNAs (out of 1205 tested) showed significantly lower expression in patients than in seronegative, parasite-negative controls, and 1 showed increased expression. There were no clear differences in miRNAs between patients in different disease stages. The miRNA profiles could not distinguish seropositive, but parasitologically negative samples from controls and results within this group did not correlate with those from the trypanolysis test. Some of the regulated miRNAs, or their predicted mRNA targets, were previously reported changed during other infectious diseases or cancer. We conclude that the changes in miRNA profiles of peripheral blood lymphocytes in human African trypanosomiasis are related to immune activation or inflammation, are probably disease-non-specific, and cannot be used to determine the disease stage. The approach has little promise for diagnostics but might yield information about disease pathology.

## Introduction

African Trypanosomiases (AT) cause devastating diseases of livestock and humans in areas of Africa that harbor the vector, the Tsetse fly. Approximately 70 million people are currently estimated to be at risk of contracting the disease. Between 2000 and 2009, about 175,000 human cases were reported, the vast majority of which were caused by *Trypanosoma brucei gambiense* in West Africa [1]. Human African trypanosomiasis (HAT) new infection rates are currently relatively low, at about 10,000 cases per year [2] but maintenance of this level relies on continuous surveillance efforts [2]. The conventional profile of human African trypanosomiasis (HAT) includes an initial hemolymphatic stage (stage I), with no specific signs [3]. This progresses to a late stage (stage II) involving the central nervous system. Progress is much slower for *T. b. gambiense* infection than for infection by the East African form, *T. b. rhodesiense*. Although most patients eventually succumb to infection if untreated, a few cases have been reported in which patients become asymptomatic or even self-cure [4], [5].

The standard serological screening method for *T. gambiense* disease is the Card Agglutination Test for Trypanosomiasis (CATT), followed by a trypanoloysis test and parasitological confirmation by microscopy. The CATT and trypanolysis tests both rely on immunoglobulins that interact, respectively, with one and three variant antigens on the surface of the trypanosomes; the trypanolysis test is more specific [6]. Microscopy can be supplemented by DNA amplification methods in the unlikely event that facilities are available [2], [7]. The only way to determine the disease stage is via examination of the cerebrospinal fluid (CSF) for trypanosomes or lymphocytes [2]. Although some molecular markers are showing promise, these too rely upon a CSF sample [8], [9]. Ultimately, the ideal solution would be a drug, which can be used to treat both stages [10], [11], but in the meantime less invasive methods to determine the disease stage would aid control efforts and might remove one barrier to patients’ willingness to seek diagnosis.

CATT-seropositive individuals without parasitological confirmation are frequently encountered in *T. b. gambiense* endemic areas (e.g. [12], [13]). Some of these individuals are also positive in the trypanolysis test, ruling out false positivity due to non-specific agglutination. Follow-up of these individuals in Guinea has shown that they can be classified into three categories: (i) those who develop HAT later were presumably in the early phase of infection); (ii) those who maintain high serological responses to the CATT (>2 years) may be asymptomatic carriers and (iii) those who later becoming negative in the CATT might have self-cured [5]. Both host and parasite variations have been implicated in this diversity in disease presentation [14], [15]. Humans respond to infection with increases in various cytokines; results from mice implicate innate, macrophage-based immune responses in protection, in addition to antibody-mediated responses to the major surface antigen, the variant surface glycoprotein [15]. A recent microarray-based study of mice infected *with T. b. brucei* (which is closely related to *T. b. rhodesiense*) confirmed activation of macrophages and several cytokine responses [16].

MicroRNAs (miRNAs) are small RNAs of about 21 nt which bind to mRNAs and regulate their stability and translation [17], [18]. In mammals, they have important roles in many processes, including inflammation, immunity and control of cell growth and differentiation [19], [20], [21]. There have therefore been numerous attempts to determine whether levels of miRNAs in peripheral blood correlate with cancer progression and circulatory disease; both the cellular and the cell-free serum fractions have been investigated [22], [23], [24], [25]. Much less attention has been paid to the roles of miRNAs in infectious disease, with most experiments focusing on intracellular pathogens, especially viruses. The few reports of host miRNA responses during parasitic disease are so far restricted to diseases caused by experimental infection by Apicomplexa: they include reports of various miRNAs whose expression was affected by infection of mice with *Plasmodium* [26], [27], and of tissue culture cells with *Toxoplasma* [28], [29] or *Cryptosporidium* [30].

We have here analysed the peripheral blood miRNA profiles of humans infected with *T. b. gambiense*, with a view to both biomarker and immune response analysis.

## Methods

### Ethical Issues

Written informed consent forms were obtained from patients and healthy individuals whose blood samples were collected and included in the present study. Blood samples were collected during a larger study for diagnostics development, within the framework of the World Health Organization control program for Trypanosomiases in West Africa (RPC 222/14.06.2007). Our study was also approved by the Heidelberg Ethical Commission (S-171/2012). All individuals who participated in the present study received an explanation of the scope of the study before they signed the consent forms.

### Blood Samples

During routine field screening by teams of the WHO control program for Trypanosomiases in West Africa, people in the Boffa sleeping sickness focus (Guinea) were screened with the CATT for whole blood. Samples with a positive CATT result were screened using the CATT plasma dilution test; all individuals that were positive at a dilution of 1∶4 or less were further examined for parasites using the buffy coat concentration technique [31], and by examination of lymph node aspirates if available, as well as a trypanolysis test [31]. Stage determination was done by white cell count for all newly infected individuals. All individuals with a positive CATT test, with or without confirmed presence of the parasite were invited to the Bofa local health center for enrollment into the surveillance program and treatment, whether or not they agreed to take part in the study. By venopuncture, 2.5 ml of blood was collected from all consented participants directly into PAXgene Blood RNA tubes (PreAnalytics-BD, New Jersey, USA). The tubes were kept at −20°C for 2 days, then at −80°C.

### Total RNA Extraction

Total RNA was extracted from blood samples using the peqGold RNA extraction reagent (PeqLab) following an optimized procedure. Blood samples in PAXgene tubes were centrifuged at 5000×g for 10 min at 4°C. The supernatant was discarded and the pellet completely re-suspended in 10 ml of nuclease-free water by vortexing. Samples were again centrifuged at 5000×g for 10 min at 4°C and the resulting pellet re-suspended in 2 ml of TriFast PeqGold. 400 µl of chloroform were added and the samples were homogenized for 30 sec at room temperature and then left for 3 min at room temperature. The aqueous phase was separated by centrifuging at 12000×g for 15 min at 4°C and transferred into an RNase-free Eppendorf tube containing 500 µl of isopropanol. The tubes were kept at −20°C for 1 h and centrifuged at 12000×g for 10 min at 4°C. The resulting RNA pellet was washed two times in 75% ethanol, re-dissolved, precipitated twice with 3 M sodium acetate and re-suspended in 50 µl of water. The quality of total RNA was checked by gel analysis using the Total RNA Nano Chip assay on an Agilent 2100 Bioanalyzer (Agilent Technologies GmbH, Berlin, Germany). RNA concentrations were determined using the NanoDrop spectrophotometer (NanoDrop Technologies, Wilmington, USA).

### Molecular Diagnosis

PCR-based diagnosis was performed on all patient samples using species-specific primers. DNA was extracted from samples by ethanol precipitation of the aqueous phase obtained after RNA extraction using peqGold Trifast following the manufacturer’s recommendations (Peqlab, Erlangen, Germany). The PCR reaction was carried out in a 25 µl reaction using the Q5 high-fidelity DNA polymerase (New England Biolabs, Frankfurt, Germany). One tenth of the DNA sample (equivalent to 0.25 ml blood) was used. The primers were for specific detection of *T. b. gambiense* [32]. Product DNA was visualized by ethidium bromide staining of a 1.5% agarose gel. Results are included in Table 1.

**Table 1:** Sample classification based on multiple diagnostic tests.

Patient	CATT	Mn-BC	Cell	Stage	PCR	Trypano-lysis	Status	miRNA pattern
Bo.470/6	+	>100	0	I	+	+	HAT	A
Bo.471/6	+	>50	0	I	+	+	HAT	A
Bo.472/6	+	+	0	I	+	+	HAT	A
Bo.475/6	+	10	5	I	+	+	HAT	A
Bo.480/6	+	>100	5	I	+	+	HAT	A
Bo.481/6	+	6	1	I	+	+	HAT	A
Bo.484/6	+	>100	1	I	+	+	HAT	A
Bo.487/6	+	+	2	I	+	+	HAT	A
Bo.502/6	+/−		1	I	+	+	HAT	A
Bo 482/6	+	>50	32	II	+	+	HAT	A
Bo.473/6	+	6	6	II	+	+	HAT	A
Bo.474/6	+	10	212	II	+	+	HAT	A
Bo.476/6	+/−	>50	541	II	+	+	HAT	A
Bo.477/6	+	>20	15	II	+	+	HAT	A
Bo.478/6	+	>10	228	II	+	+	HAT	A
Bo.479/6	+	2	13	II	+	+	HAT	A
Bo.≈/6	+	>100	80	II	+	+	HAT	A
Bo.485/6	+	>50	6	II	+	+	HAT	A
Bo.486/6	+	+	6	II	+	+	HAT	A
Bo.488/6	+	>100	51	II	+	+	HAT	A
Bo.492/6	+	−			−	+	Seropo/AT	A
Bo.494/6	+	−			−	−	Seropo/AT	A
Bo489/6	+	−			−	+	Seropo	A
Bo500/6	+/−	−			−	+	Seropo	B
Bo.490/6	+	−			−	+	Seropo	A
Bo.498/6	+	−			−	+	Seropo	B
Bo.527/6	+	−			−	+	Seropo	B
Bo.491/6	+	−			−	−	Seropo	A
Bo.493/6	+	−			−	−	Seropo	B
Bo.499/6	+	−			−	−	Seropo	A
Bo.520/6	+	−			−	−	Seropo	B
Bo495/6	+	−			−	−	Seropo	B
Bo.537/6	−	−			−	−	Control	B
Bo.538/6	−	−			−	−	Control	B
Bo.509/6	−	−			−	−	Control	B
Bo.511/6	−	−			−	−	Control	B
Bo.514/6	−	−			−	−	Control	B
Bo.518/6	−	−			−	−	Control	B
Bo.521/6	−	−			−	−	Control	B
Bo.529/6	−	−			−	−	Control	B

The patient codes are shown on the left. Mn-BC: Buffy coat mini concentration column, number of parasites; “+” means present but not counted; Cell: white cell count in CSF for staging; PCR: presence of parasite DNA; Trypanolysis: positive result from the trypanolysis test; Status: Ser+ − positive by CATT; AT: previously treated patient. miR expression pattern: A = more similar to infected, B = more similar to control.

### miRNA Expression Profiling

Analysis of the differential expression of circulating miRNAs was done using the miRNA Microarray System with miRNA Complete Labeling and Hyb Kit (which represents 1205 human and 144 human viral miRNAs) (Agilent) following the manufacturer’s instructions. Briefly, after total RNA extraction and quality control using the Agilent Bioanalyzer, 100 ng of total RNA was dephosphorylated using calf intestinal alkaline phosphatase at 37°C for 30 min. The samples were then denatured in 100% DMSO at 100°C for 5 min and ligated to Cyanine3-pCp at 16°C in a circulating water bath for 2 h and purified on a micro bio spin column. The eluate was vacuum dried at 55°C. Samples were resuspended in 18 µl of nuclease-free water. 4.5 µl of the 10X GE Blocking Agent and 22.5 µl of 2x Hi-RPM hybridization buffer were added to each sample and mixed by vortexing. Samples were then heated at 100°C for 5 min and kept on ice. Hybridization was done in a SureHyb chamber at 55°C for 20 h in a hybridization oven. Slides were washed two times at room temperature and once at 37°C for 5 min and scanned using an Agilent scanner (SureScan). Data was extracted using Agilent feature extraction software and analyzed with Chipster microarray data analysis software [33]. Each slide has 8 chambers. In each case, three chambers were used for control samples and the remaining five were used for individual patient or seropositive samples. All signals were measured relative to the average from the three controls. Each patient or seropositive sample was analysed once, since there was insufficient material for replicates.

### qRT-PCR

qRT-PCR was carried out to confirm the profiles observed from miRNA expression profiling. To this end, 0.75 µg of total RNA were reverse transcribed into cDNA in a total volume of 20 µl using the miScript reverse transcription kit (Qiagen, Hilden, Germany) according to the manufacturers recommendations. Following cDNA synthesis, the resulting cDNA was diluted 10-fold before being used for real time PCR. The miScript primer assay for Syber green-based real time PCR (Qiagen) was used for qRT-PCR in a total volume of 12 µl, containing 1 µl of diluted cDNA in a LightCycler 480 system (Roche, Mannheim, Germany). The entire reaction was composed of 40 cycles, consisting of an initial activation step at 95°C for 15 min followed by 40 consecutive cycles of 94°C for 15 sec, 55°C for 30 sec and 70°C for 30 sec for transcript quantification. The U1RNUB6 gene (Qiagen) was used as a standard.

### Gene Expression Profiling

Gene expression profiling was performed using the illumina Human Sentrix-12 BeadChip arrays, which contain more than 47,000 probes (Life Technologies, Darmstadt, Germany). Biotin-labeled cDNA samples were prepared according to Illumina's recommended sample labeling procedure [34]. In brief, 200 ng total RNA was used for complementary DNA (cDNA) synthesis, followed by an amplification/labeling step (*in vitro* transcription) to synthesize biotin-labeled cRNA according to the Illumina Total Prep RNA Amplification Kit (Life Technologies). Biotin-16-UTP was purchased from Roche Applied Science (Penzberg, Germany). The cRNA was column purified and eluted in 60 µl of water. The quality of cRNA was checked using the RNA Nano Chip Assay on an Agilent 2100 Bioanalyzer and spectrophotometrically quantified (NanoDrop). Hybridization was performed at 58°C in GEX-HCB buffer (Life Technologies) at a concentration of 100 ng cRNA/µl, in a wet chamber for 20 h. For each array, a single patient RNA was compared with pooled RNA from the controls; six individual patient samples were studied, each on a single array. Sample amounts were insufficient for replicates. Spike-in controls for low, medium and highly abundant RNAs were added, as well as mismatch control and biotinylation control oligonucleotides. Microarrays were washed once in High Temp Wash buffer (Life Technologies) at 55°C and then twice in E1BC buffer (Life Technologies) at room temperature for 5 min; in between the washing steps, they were always rinsed with ethanol at room temperature. After blocking for 5 min in 4 ml of 1% (wt/vol) Blocker Casein in phosphate buffered saline (PBS) Hammarsten grade (Pierce Biotechnology, Rockford, USA), array signals were developed by a 10-min incubation in 2 ml of 1 µg/ml Cy3-streptavidin (Amersham Biosciences, Buckinghamshire, UK) and 1% blocking solution. After a final wash in E1BC, the arrays were dried and scanned. Microarray scanning was done using an iScan array scanner (Illumina). Data extraction was done for all beads individually, and outliers with a median absolute deviation >2.5 were removed. All remaining data points were used for the calculation of the mean average signal for a given probe, and standard deviation for each probe was calculated.

Gene functions were annotated using the GeneCard database (http://www.genecards.org/) [35].

### Target Prediction and Core Analysis

MiRNA target prediction was done using the target prediction software incorporated into the Ingenuity Pathway Analysis (IPA) software Ingenuity Systems, www.ingenuity.com. To this end, both highly predicted and experimentally identified miRNA targets with relevance to pathogen induction as well as immune responses were queried. All resulting miRNA targets were scored against all genes that were differentially regulated from the gene expression profiling experiments. miRNAs and corresponding targets that went through this filter were subjected to a core analysis in IPA to find out cross relationships and potential downstream effects involving other molecules that could be major players in infection.

### Statistical Analysis

Data analysis was done using Chipster microarray data analysis software [33]. All samples were quintile normalized across chips and filtered according to standard deviation (0.95) and interquartile range. The empirical Baye’s two group t-test (p<0.05) was used to test for differential miRNA expression between different sample groups. The Benjamini-Hochberg correction was applied to all p-value calculations. For linkage clustering, the Pearsons correlation coefficient was calculated. Quantitative real-time (qRT-) PCR was carried out in triplicates for a confirmation of microarray data. Resulting data were expressed as mean ± standard deviation (SD). All miRNA with a mean difference having a p-value of <0.05 for a two-sided unpaired student *t-test* were considered significantly regulated.

## Results

### Patient Screening

During a screening campaign, 14,445 individuals were screened with the CATT test. 324 tested positive for the CATT on whole blood while 114 had a positive test for the CATT using plasma at a fourfold dilution. Trypanosomes were found in 45 of the latter; the remaining 69 subjects were classified as seropositive, parasite-negative. 40 samples were chosen for our study (Table 1). We included 8 control samples from sero-negative, parasite-negative people (group C). A second group of CATT-positive, but parasitologically and PCR-negative individuals (group CP) included 5 who were trypanolysis-positive, and 7 who were trypanolysis-negative. The remaining 20 subjects were patients who were positive by CATT, PCR and parasite detection: 9 in stage-I (group HAT-1), and 11 in stage-II (group HAT-2). We note that the parasitological test used here is very sensitive, detecting 10 parasites/ml blood when 5 ml blood is used as starting material [31]; the PCR test that we performed, using DNA from 0.25 ml blood, had a similar sensitivity of 10 trypanosomes/ml [32]. The concordance of these results can be seen in Table 1. RNA was prepared from the 40 samples and used for gene expression analysis.

### miRNA Expression Analysis

**Figure 1:**
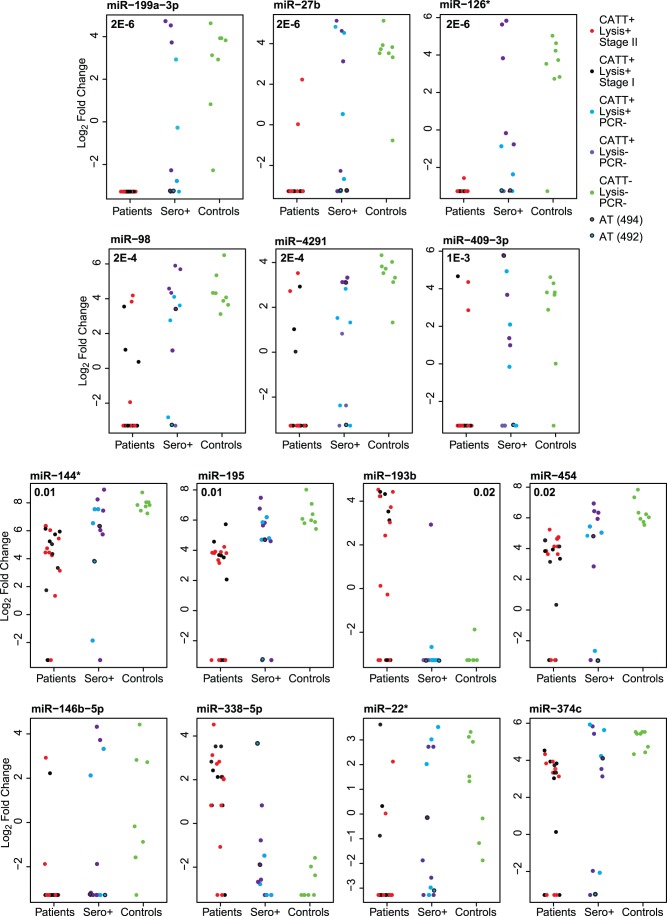
miRNAs with altered abundance in sleeping sickness. Data for the miRNAs from Table 1 are illustrated, showing the Log2 fold changes for individual patients. The color code for the spots is at top right.

We analyzed the expression levels of 1205 miRNAs. Results are accessible at ArrayExpress (http://www.ebi.ac.uk/arrayexpress/) under accession number E-MTAB-1467. Fourteen miRNAs were found to be differentially expressed between all patients (groups HAT-1 and HAT-2) and the control group (group C) (Table 2, Figure 1). Among these 14 miRNAs, 13 were significantly differentially regulated between patients with stage-II disease (group HAT-2) and controls (group C) while ten miRNAs were differentially expressed between stage-I patients (group HAT-1) and controls (group C)**.** However, not one miRNA could be used to distinguish between stage I (HAT-1) and stage II (HAT-2) patients.

**Table 2:** miRNAs with altered abundance in sleeping sickness.

miRNA ID	Log_2_FC stage I	p-value stage I	Log_2_FC stage II	p-value stage II	Log_2_FC all	p-value all
miR-199a-3p	−6.9	2 E-4	−6.9	1E-5	−6. 8	2E-6
miR-27b	−6.8	2E-4	−6.7	6E-5	−6.6	2E-6
miR-126*	−6.7	3E-4	−6.4	2E-4	−6.4	2E-6
miR-98	−5.9	3E-3	−6.3	6E-4	−6.1	2E-4
miR-409-3p	−5.5	7E-3	−5.1	0.01	−5.2	1E-3
miR-4291	−5.3	7E-3	−6.1	6E-4	−5.6	2E-4
miR-146b-5p	−4.5	0.03	−4.3	0.02	n/a	n/a
miR-454	−4.1	0.04	−4.1	0.04	−4.1	0.02
miR-193b	4.4	0.04	4.1	0.05	4.2	0.02
miR-195			−5.1	0.01	−4.5	0.01
miR-144*			−4.7	0.01	−4.3	0.01
miR-22*			−5.1	3E-3	n/a	n/a
miR-374c			−4.6	0.02	n/a	n/a
miR-338-5p	4.7	0.01			n/a	n/a

All miRNAs that showed some alteration in at least one stage of sleeping sickness are shown. Log2 FC is Log2 of the arithmetic mean fold change in patients relative to the average value for controls. The p-values are also shown. Student’s t-tests were used to compare paired and multiple groups, with a Benjamini-Hochberg correction for false discovery. A threshold of 0.05 was set for significance. Although some miRNAs were significantly altered in only one stage, further analysis of these showed no significant difference between stage I and stage II.

**Figure 2:**
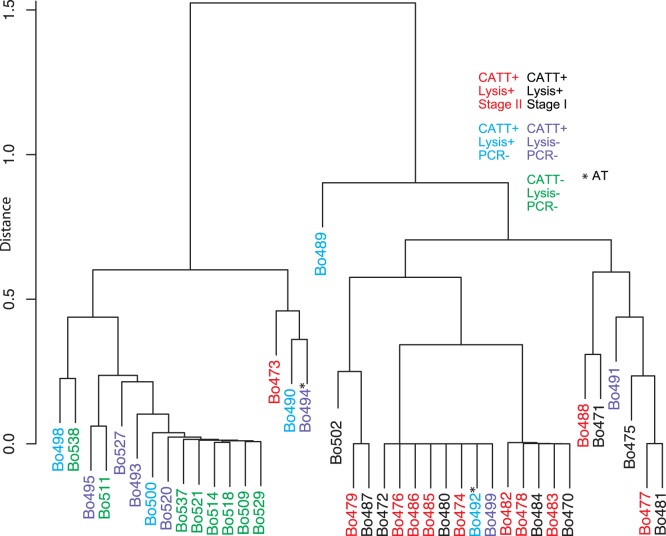
Cluster dendrogram for all samples. The samples were classified according to miRNA expression patterns, using the miRNAs in Table 1, and a dendrogram was made to show the relationships. The color codes are shown on the Figure.

Of the 14 miRNAs, miR-193b and miR-338 were increased in patients, the others were decreased. Three individual miRNAs (miR-199a-3p, miR-27b and miR-126*) were able to differentiate all patients from controls (group C) (p<0.05) (Figure 1 & Figure 2). However, in each case, at least one seropositive, trypanolysis-negative person also showed a “patient-like” miRNA level and in one case (mir-126*) an uninfected control also had a patient-like level. To confirm the results, the three miRNAs were analyzed by qPCR of 16 patient and 8 control samples. For miR-199a-3p and mir27b, the average differences were only 2-fold (p-values 0.03 and 0.01 to distinguish between patient (HAT) and control (C)). In contrast, the patients had, on average, 8-fold less mir-126* than controls (p = 5E-10).

The CATT-positive, but parasite- and PCR-negative patients (group CP) showed a range of miRNA profiles, which did not correlate with the results of the trypanolysis test (Figure 1). We were interested to see whether or not the miRNA profiles of the seropositive group could be used to predict a possible infection in these subjects. First, we applied two-group and multiple group tests to the three sample groups. The group included two patients that had been treated and had returned for follow-up. One was trypanolysis-negative, the other positive. Unfortunately, we have no information about the interval between treatment and sampling for these two individuals. Both of these samples showed an infected-like miRNA profile (Table 1). For the six miRNAs with the best correlation with infection, the trypanolysis-positive treated patient consistently showed an infected-like pattern, whereas the trypanolysis-negative patient did not (Figure 1). The remaining group CP samples split equally between the infected-and uninfected-like patterns. Of the five trypanolysis-positive samples in group CP, two had infected-like patterns, while three resembled the controls; exactly the same was seen for the trypanolysis-negative samples. Next, we created a dendrogram by treating the levels of the differentially regulated miRNAs as individual traits. Some of the group CP samples indeed clustered together with those from patients, but again there was no correlation with the positive trypanolysis result. This suggests that the miRNA profiles we observed might not be specific to trypanosomiasis alone, but could also result from other conditions.

### Gene Expression Profiling

To obtain a preliminary idea of whether mRNAs that are miRNA targets were also affected by HAT, six of the patient samples were subjected to gene expression profiling, using various pools of three CATT-negative sera as controls. In total, 656 genes were found to be significantly (p<0.05) differentially regulated between patients and controls, but only 56 were more than 2-fold decreased and none was more than 2-fold increased (Table S1). There was no difference between stage-I and stage-II patients for any of these RNAs. Since we have no data on variations within the controls, it is not possible to tell whether any of the gene expression changes really is characteristic of HAT.

Only 34 of the genes with two-fold reduced mRNA are functionally annotated, and they are involved in very diverse functions. They include RFXAP, encoding a transcriptional activator for some MHC class II genes; EFNAH4, a protein tyrosine kinase potentially involved in the regulation of erythropoiesis and nervous system gene expression; LILRB5 and LILRA4, which encode members of a leucocyte immunoglobulin-like receptor family; and CARD6, a signal transduction regulator that may affect the function of the transcription factor NF-kappaB. Further analysis would be required to find out whether these changes are specific to HAT.

### miRNA Target Prediction and Core Analysis

We now predicted targets for the nine miRNAs that showed some difference between all patients and controls, and also for miR-195 as the next best-scoring miRNA (stage II only). More than 3,000 highly predicted or experimentally investigated putative targets were found. Next, we looked to see whether any of the genes showing changed mRNA levels could also be a possible target of the ten miRNAs. Results are shown in Table S2. Since cell populations were used, we do not know whether miRNAs and their cognate mRNAs were expressed in the same cells, so we cannot claim any causative relationships. However, if miRNAs were expressed in the same cell, it would be expected (if anything) to decrease the abundance of target mRNAs.

Of the 34 predicted targets, only one, RFXAP, was down-regulated more than 2-fold at the level of steady-state mRNA, but the cognate miRNA was decreased as well. TIMP2, a moderately elevated mRNA encoding a metalloprotease inhibitor, is a possible target of two of the down-regulated miRNAs (miR-4291 and miR-454). Among the genes with mildly decreased expression, four (GPR146, EIF2S1, PLA2G4D and MAPK10) were possible targets of one up regulated miRNA (miRNA-193b). One only regulated cytokine gene that was a potential target showed only a very small change and encoded CXCL11.

## Discussion

In this study, we have identified nine miRNAs whose levels were altered in the peripheral blood of HAT patients. When, however, we compared the patient miRNA profiles with those of subjects who were CATT-positive, but PCR-negative, we discovered that some of the latter, too, had “HAT-like” miRNA profiles. Moreover, such profiles were even seen in trypanolysis-negative samples. While it is conceivable that these people had been infected with trypanosomes that had low, or no, expression of the antigens detected in the trypanolysis test [6], or that our PCR had a lower sensitivity than that published [36], this is rather unlikely. Alternatively, it might be that people with very low (undetectable) parasite loads, who were able to control the infection, show miRNA profiles resembling those of the uninfected controls. However, the simplest interpretation is that the miRNA changes that we observed in HAT patients were non-specific and perhaps related to immune activation or inflammation. Indeed, non-specific activation might explain some of the positive CATT results from parasite-negative samples. Unfortunately, also, none of the miRNAs that we identified could distinguish between stage I and stage II infection.

During HAT, high immunoglobulin and immune complex levels are documented in humans for both peripheral blood and the CSF; peripheral polyclonal lymphocyte activation and changes in B- and T-cell populations were also seen [37], [38], [39], [40]. The miRNA and mRNA transcriptomes of peripheral blood cells reflect changes in cell types present, as well as in the physiology of those cells. Using our limited sample, we did not see any transcriptome changes that correlate with known pathology. Some of the miRNA changes, however, did show potential links with cytokines or cell proliferation.

miR-199a-3p, miR-193b and miR-126 have all been implicated in the suppression of cell proliferation [41], [42], [43], [44], [45], [46], [47], [48], [49], [50], [51]. We speculate, therefore, that the decreases in miR-199a-3p and miR-126 that we observed in our HAT samples could be related to an increase in leukocyte proliferation. miR-193b, however was the only reproducibly increased miRNA, which does not fit with this hypothesis.

Elevated interferon gamma levels have been seen in both *T. gambiense* [52], [53] and *T rhodesiense* [54] patients. Increases in TNF alpha have been seen in *T. gambiense* patients [53], [55], [56] and in vervet monkeys infected with *T. rhodesiense* [57]. It is therefore interesting that miR-144* was decreased in most HAT patients, since miR-144* has been reported to be involved in the inhibition of TNF-alpha and IFN-gamma production and T-cell proliferation [58].

Mir27b is enriched in liver cells, is a negative regulator of several mRNAs involved in lipid metabolism [59], and is also involved in the control of angiogenesis [60]. An increase in miR27b is required for expression if inducible nitric oxide synthase (iNOS) during infection of epithelial cells by *Crytosporidium parvum*: miR27b decreases expression of a negative regulator [61], [62]. In contrast, mir27b decreased in HAT patient peripheral blood.

In conclusion, we have shown that *T gambiense* infection of humans causes alterations in the expression of miRNA in peripheral blood leucocytes. Unfortunately, however, no miRNA could be specifically linked to HAT infection or used to predict the stage of the disease. Instead, several of the strongly affected miRNAs have been linked previously to changes in cellular proliferation, which might reflect the lymphocyte activation that is seen in the disease.

## Supporting Information

Table S1 mRNAs with significantly altered abundance in sleeping sickness patients. Sheet 1: Results for individual patients are shown, with a corresponding result for pooled controls. Sheet 2: Annotations from GeneCARD for the regulated genes.
(XLSX)

Table S2 Predicted targets of the miRNAs that were significantly changed in sleeping sickness samples. Only mRNAs that also showed a change in expression are included.
(XLS)
