# Correction: The miRNA and mRNA Signatures of Peripheral Blood Cells in Humans Infected with *Trypanosoma brucei gambiense*

**DOI:** 10.1371/annotation/b13cc66f-93c2-4582-99c1-359276235acc

**Published:** 2013-07-11

**Authors:** Smiths Lueong, Gustave Simo, Mamadou Camara, Vincent Jamonneau, Jacques Kabore, Hamidou Ilboudo, Bruno Bucheton, Jörg D. Hoheisel, Christine Clayton

The name of the first author was spelled incorrectly. The correct name is: Smiths Lueong. The correct citation is: Lueong S, Simo G, Camara M, Jamonneau V, Kabore J, et al. (2013) The miRNA and mRNA Signatures of Peripheral Blood Cells in Humans Infected with Trypanosoma brucei gambiense. PLoS ONE 8(6): e67312. doi:10.1371/journal.pone.0067312. 

